# Monitoring the volatile language of fungi using gas chromatography-ion mobility spectrometry

**DOI:** 10.1007/s00216-021-03242-6

**Published:** 2021-03-06

**Authors:** Verena Speckbacher, Susanne Zeilinger, Stefan Zimmermann, Christopher A. Mayhew, Helmut Wiesenhofer, Veronika Ruzsanyi

**Affiliations:** 1 Department of Microbiology, Leopold-Franzens-Universität, 6020 Innsbruck, Austria; 2 Institute of Electrical Engineering and Measurement Technology, Leibniz Universität Hannover, 30167 Hannover, Germany; 3 Institute for Breath Research, Leopold-Franzens-Universität Innsbruck, Innrain 66, 6020 Innsbruck, Austria; 4 Tiroler Krebsforschungsinstitut (TKFI), Innrain 66, 6020 Innsbruck, Austria

**Keywords:** *Trichoderma atroviride*, *Fusarium oxysporum*, Ion mobility spectrometry (IMS), Secondary metabolites, Microbial volatile organic compounds (MVOCs), Fungi, Mycoparasitism, 2-Octanone, Light response

## Abstract

*Fusarium oxysporum* is a plant pathogenic fungus leading to severe crop losses in agriculture every year. A sustainable way of combating this pathogen is the application of mycoparasites—fungi parasitizing other fungi. The filamentous fungus *Trichoderma atroviride* is such a mycoparasite that is able to antagonize phytopathogenic fungi. It is therefore frequently applied as a biological pest control agent in agriculture. Given that volatile metabolites play a crucial role in organismic interactions, the major aim of this study was to establish a method for on-line analysis of headspace microbial volatile organic compounds (MVOCs) during cultivation of different fungi. An ion mobility spectrometer with gas chromatographic pre-separation (GC-IMS) enables almost real-time information of volatile emissions with good selectivity. Here we illustrate the successful use of GC-IMS for monitoring the time- and light-dependent release of MVOCs by *F. oxysporum* and *T. atroviride* during axenic and co-cultivation. More than 50 spectral peaks were detected, which could be assigned to 14 volatile compounds with the help of parallel gas chromatography-mass spectrometric (GC-MS) measurements. The majority of identified compounds are alcohols, such as ethanol, 1-propanol, 2-methyl propanol, 2-methyl butanol, 3-methyl-1-butanol and 1-octen-3-ol. In addition to four ketones, namely acetone, 2-pentanone, 2-heptanone, 3-octanone, and 2-octanone; two esters, ethyl acetate and 1-butanol-3-methylacetate; and one aldehyde, 3-methyl butanal, showed characteristic profiles during cultivation depending on axenic or co-cultivation, exposure to light, and fungal species. Interestingly, 2-octanone was produced only in co-cultures of *F. oxysporum* and *T. atroviride*, but it was not detected in the headspace of their axenic cultures. The concentrations of the measured volatiles were predominantly in the low ppbv range; however, values above 100 ppbv were detected for several alcohols, including ethanol, 2-methylpropanol, 2-methyl butanol, 1- and 3-methyl butanol, and for the ketone 2-heptanone, depending on the cultivation conditions. Our results highlight that GC-IMS analysis can be used as a valuable analytical tool for identifying specific metabolite patterns for chemotaxonomic and metabolomic applications in near-to-real time and hence easily monitor temporal changes in volatile concentrations that take place in minutes.

## Introduction

Microbial volatile organic compounds (MVOC) are chemically very diverse primary or secondary metabolites, which are easily transported in the air. Depending on the environmental conditions, microbes produce a plethora of compound- and even strain-specific MVOCs [1–3], which play a significant role in microbial interactions [4–6], including signaling between inter-species and inter-kingdom interactions [6–8]. MVOC biosynthesis depends strongly on physiological conditions, developmental stages, and environmental cues, thereby resulting in a characteristic MVOC profile emitted by pure cultures in a reproducible way under standardized conditions. These specific profiles therefore enable an on-line real-time characterization of the organisms through temporal measurements of the released MVOCs [9–12].

*Trichoderma atroviride* is a cosmopolitan, soil-inhabiting, filamentous fungus belonging to the division of *Ascomycota*. *T. atroviride* is able to promote plant growth and induce systemic resistance in plants against biotic and abiotic stresses. Furthermore, it is a mycoparasite antagonizing a broad range of plant pathogenic fungi by direct necrotrophic parasitism, leading to its frequent application as a biologic control agent in agricultural plant protection and pest control [13–15]. Typically attacked phytopathogenic host fungi, such as *Rhizoctonia solani* and *Fusarium oxysporum*, are causing worldwide multi-billion dollar crop losses and result in huge investments for chemical pesticides every year [16], the use of which is detrimental to the environment and to human health. Thus, there is a high demand for sustainable biological solutions. Efforts need to be intensified to gain in-depth knowledge on the underlying microbial interactions [15, 17].

One technique, which allows an on-line determination of a high number of different volatiles in a complex chemical matrix, is gas chromatography coupled to ion mobility spectrometry (GC-IMS). The advantages of this method for the current application are its good sensitivity (low ng/L range) for selected volatiles and the direct on-line introduction of the sample gas. Thus, no time-consuming sample preparation steps are required that could result in the loss of the analytes. Furthermore, it facilitates an almost real-time analysis of the volatiles allowing the monitoring of metabolic changes with a time resolution of several minutes. The comparatively small size of the device also makes this technique attractive for field applications.

Some pilot studies have highlighted the use of GC-IMS to detect MVOCs for indoor air quality control [18, 19]. These studies were focused on the recognition of specific mold-emitting volatiles, which are hazardous to human health, in order to allow the recognition, localization, and elimination of these fungi.

The aim of our study is to demonstrate the use of GC-IMS to sample the headspace above the filamentous fungi *F. oxysporum* and *T. atroviride* directly without any sample preparation and to monitor how MVOC concentrations change in almost real time as a function of time and cultivation conditions. Another aim is to test the feasibility of the method for identifying the fungal plant pathogen *F. oxysporum* as well as the mycoparasitic fungus *T. atroviride* by their characteristic MVOC profiles for chemotaxonomic and metabolomic applications.

## Methods

### Strains and culture conditions

The mycoparasitic fungus *Trichoderma atroviride* IMI 206040 (hereafter assigned IMI) and the plant pathogenic fungus *Fusarium oxysporum f. sp. lycopersici* (strain 4287; hereafter assigned Fo) were used in this study. Fungi were cultivated in petri dishes (94 × 16 mm, Greiner Bio-One GmbH, Kremsmünster, Germany) containing 25 ml potato dextrose agar (PDA; Becton, Dickinson and Company, Le Pont De Claix, France).

For pre-cultivation, an agar piece containing mycelia from the stock cultures kept at 4 °C was inoculated upside-down in the center of an agar plate. Inoculated plates were incubated in a climate chamber at 25 °C applying a light-dark cycle (12 h:12 h; 383 Lux; Snijders Micro Clima-Series TM Labs Economic Lux Chamber; Snijders Labs, Tilburg, Netherlands) for 1.5 days. To reach exponential growth, a 6-mm diameter agar plug of the actively growing fungal colony margin was propagated at least twice after 1.5 days each upside-down to the center of a fresh agar plate.

For GC-IMS analysis, a 6 mm diameter agar plug of the actively growing colony margin from the final pre-culture was picked upside-down and placed at the edge of the bottom of a 150 ml glass flask (Duran GmbH, Mainz, Germany) containing 25 ml PDA for either axenic culture or co-culture. IMI and Fo were pre-grown at 25 °C for 20 h and 44 h, respectively, before the flasks were placed into the incubator for headspace sampling. For the co-cultures, the mycoparasite and the host fungus were placed on the agar surface on opposite sides. The headspace of PDA medium (without fungal inoculation) was also investigated. The glass bottles were closed with Teflon® screw caps (Bohlender™, Merck, Vienna, Austria) with two openings, one as an air inlet and the other acting as an air outlet. The influence of exposure to light was investigated for all samples by using two different light regimes: a) flasks were covered with several layers of aluminum foil to ensure dark growth conditions (DD); and b) flasks were exposed to light using a lamp (LED Lumistixx®, OSRAM, Germany) attached to the internal wall of the oven twice a day for 2 h each time (light-dark; LD).

### Headspace measurements

#### Sampling during cultivation

For headspace measurements, the cultures in glass bottles were held in an incubator and connected in a gastight way, parallel to each other [20]. To avoid condensation, the inner temperature of the oven (and hence the temperature of the headspace air) was held at 40 °C, while the water bath was kept at 23 ± 2 °C. For ventilation, purified air at 5 ml/min was continuously streaming through the flasks, regulated by four mass flow controllers (MCF, Bronkhorst, Ruurlo, Netherlands). Additionally, 5 ml/min of purified air was connected as a dilution flow to the flasks to reduce moisture in the samples. Headspace air samples were collected in the incubator at 40 °C in 100 ml and 250 ml glass syringes (Socorex, Ecublens, Switzerland). Sampling and measurement were performed twice a day at 21, 24.5, 43.5, 48.5, 68.5, 72.5, 91.5, and 96.5 and a final measurement at 115.5 h after inoculation. All experiments were repeated using four biological replicates.

#### GC-IMS analysis

A high-resolution (*R* = 90) GC-IMS, developed at Leibniz University of Hannover, was applied to monitor the emitted MVOCs. A detailed description of the system can be found in [21]. Brief details are provided here, which are pertinent to this study. Samples were injected immediately after collection through a heated perfluoroalkoxy (PFA) inlet tube of 1/16 in. in diameter (40 °C) into the GC column using a stainless-steel sample loop (200 μl) installed on a six-way valve (VICI AG International, Schenkon, Switzerland). Volatiles were separated using a RTX volatiles column (10 m × 0.53 mm × 2 μm, Restek GmbH, Bad Homburg, Germany) working at a constant temperature of 50 °C. The carrier-gas-flow-rate program was as follows: 3 ml/min for 10 min and then 10 ml/min for another 10 min, resulting in a total GC-runtime of 20 min. The IMS has a drift tube length of 7.5 cm and a drift voltage of 5 kV was applied. The instrument was operated at 40 °C, using purified air as the drift gas at the flow of 150 ml/min and 10 mbar above the ambient pressure. A radioactive β-emitter ^3^H (300 MBq) was used as the ionizing source.

GC-IMS spectra were analyzed using the software LAV (version 2.2.1, GAS mbH, Dortmund, Germany). The volume intensities of the detected spectral peaks were calculated and used for quantification.

The headspaces of axenic and co-cultures of IMI and Fo, grown under LD or DD conditions, were monitored for the emission of MVOC twice a day with four independent biological replicates taken each time, in order to obtain a measure of the reproducibility of the method. The mean and associated standard deviation of concentrations of the selected MVOCs were calculated for every sampling time point and cultivation condition and plotted to show reproducibility at each time point and to visualize the characteristic trend during cultivation and/ or differences in the trends between axenic cultures of IMI and Fo or their co-cultures [20].

The combination of retention and drift time measurements provide additional analytical capabilities over just using GC-MS or IMS, which enhance the confidence in correctly assigning the volatile compounds for near to real-time measurements once the GC-IMS is calibrated.

#### GC-IMS spectral peak identification

To identify the volatiles in the GC-IMS spectra with a high level of confidence, GC-MS analyses of the headspace were also undertaken. Volatiles were extracted using needle traps (NT; PAS Technology, Magdala, Germany) containing 2 cm of Carbopack X and 1 cm of Carboxen 1000 sorption materials. This method of sampling is necessary, because the volatiles are in the low-ppb range and hence need preconcentration before measuring. The NT was connected to the 250-ml glass syringe filled with 200 ml of the sample by piercing the membrane-containing adapter and was placed into an incubator at 40 °C to ensure constant temperature during the extraction process. The other end of the NT was connected to an electronic mass flow controller (model F-201DVRAD-11-V, Bronkhorst, Ruurlo, Netherlands) via a Teflon tube. For the generation of sample flow, a pump (Vacuubrand, Wertheim, Germany) was placed at the end of the sampling system. A total flow of 8 ml/min was used through the NT during adsorption. MVOCs were released from the sorbents by direct thermal desorption of the NT at 290 °C using splitless mode in the heated injector of a 7890A gas GC equipped with a 5975C Inert XL mass selective detector (both from Agilent Technologies, Waldbronn, Germany). A capillary column RXT-624 30 m × 0.32 mm × 1.8 μm (Restek Corporation, U.S., Bellefonte, PA, USA) was used. The oven temperature program was set to 40 °C for 0 min, then 5 °C/min to 150 °C for 2 min, and then 10 °C/min to 240 °C for 5 min.

MS analyses were performed in full scan mode, with a scan range of *m/z* 20–200. Ionization of the separated compounds was done by electron impact at 70 eV. Chromatographic data were acquired using the Agilent Chemstation Software (GC-MS Data Analysis from Agilent, Waldbronn, Germany) and analyzed using the software OpenChrom (vers. 1.4.0, Lablicate GmbH, Hamburg, Deutschland). The mass spectrum library NIST 2008 (Gatesburg, USA) was applied for identification. Following identification of the emitted volatiles by GC-MS, each volatile was measured separately with GC-IMS to obtain the reference data (drift and retention times).

#### Calibration of GC-IMS

Multi-compound calibration mixtures were prepared from pure liquid substances using a liquid calibration unit (LCU, Ionicon Analytik GmbH, Innsbruck, Austria), by nebulizing the liquid solutions prepared from the selected substances in water in the evaporation chamber of the device at 80 °C and diluted to the required concentrations. The reference substances (see Table 1) with purities ranging from 98 to 99.9% were purchased from Sigma-Aldrich (Austria). The calibration of the device for the compounds was performed using a concentration range of 1 to approx. 100 ppbv, and, if required, such as for 2-heptanone, to 500 ppbv. Using an internal pump from a by-pass flow, the continuous sample flow from the LCU was introduced into the sample loop of the GC-IMS via the heated PFA inlet tube. Calibration curves were obtained based on the 3-fold analyses of five different volatile concentrations.

**Table 1:** Compounds and characteristic GC-IMS retention times, reduced ion mobilities (*K*_0_), dynamic ranges, detection limits (LODs), and relative standard deviations (RSDs) of the identified volatile compounds

Compound	CAS number	Retention time [s]	*K*_0_ [cm^2^ V^−1^ s^−1^]		RSD%	LOD [pptv]	Dynamic range [ppbv]
			Peak 1	Peak 2			
Ethanol	64-17-5	42	1.890	1.739	4.38	138	0.43–70
Acetone	67-64-1	49	1.919	1.759	1.24	23	0.07–100
1-Propanol	71-23-8	56	1.786	1.560	2.42	47	0.14–100
Ethylacetate	141-78-6	74	1.800	1.471	3.72	13	0.40–50
2-Methyl-propanol	78-83-1	78	1.689	1.428	1.53	139	0.50–70
3-Methyl-butanal	590-86-3	95	1.580	1.391	1.7	193	0.60–100
2-Pentanone	107-87-9	118	1.738	1.435	1.77	66	0.20–100
3-Methyl-butanol	123-51-3	150	1.585	1.300	4.60	149	0.45–100
2-Methyl-1-butanol	137-32-6	152	1.581	1.306	5.80	106	0.32–100
1-Butanol-3-methyl-acetate	123-92-2	450	1.512	1.116	3.53	32	0.10–70
2-Heptanone	110-43-0	518	1.577	1.211	0.86	51	0.15–200
1-Octen-3-ol	3391-86-4	670	1.696	–	4.51	78	0.24–100
3-Octanone	106-68-3	686	1.485	1.131	2.47	22	0.07–100
2-Octanone	111-13-7	696	1.445	1.102	2.52	30	0.09–100

## Results

**Fig. 1:**
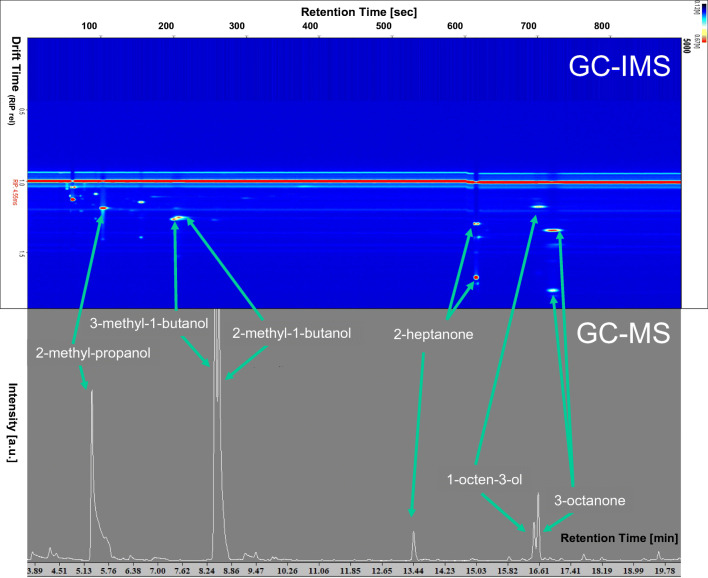
Illustrative GC-MS and GC-IMS chromatograms showing the MVOCs detected in the headspace of an axenic *T. atroviride* culture after 72 h of incubation under LD conditions

Figure 1 provides an example of GC-IMS and GC-MS chromatograms, with the key volatiles being identified, namely 2-methyl-propanol, 3-methyl-1-butanol, 2-methyl-1-butanol, 2-heptanone, 1-octen-3-ol, and 3-octanone detected in the headspace of an axenic *T. atroviride* culture after 72 h of incubation under LD conditions. Note the differences in the retention times between GC-IMS and GC-MS, with the shorter retention times in the former allowing near to real-time measurements.

### GC-IMS calibration results

Fourteen MVOCs were identified with a high level of confidence in the headspace of the fungal cultures. Of these compounds, we could detect 1-propanol at 12.8 ± 5.3 ppb, ethylacetate at 0.3 ± 0.1 ppb, and ethanol at 24.4 ± 11.0 ppb in the headspace of the uninoculated growth medium during the same incubation conditions and period. For almost all of the compounds, two peaks could be detected at the same retention time, which correspond to monomer and dimer product ions. The formation of dimer peaks is concentration dependent; for 1-octen-3-ol, only the monomer could be observed, presumably because the concentrations were too low to permit dimer formation. Temporal variations in concentrations of the identified compounds during cultivation of IMI and Fo, either in axenic culture or in co-culture, will be discussed in detail. Table 1 summarizes the results from the GC-IMS calibration measurements, including retention times, reduced ion mobilities, the dynamic ranges, the calculated limits of detection (LOD), and relative standard deviations (RSDs). The variability of the drift and retention times were 0.9–1.2% and 0.5–3.8%, respectively. The LOD was calculated according to $\frac{\mathrm{SD}}{b}$ *k*_*n*_, where SD is the standard deviation of the blank, *b* is the slope of the calibration line (near to the LOD, since in several cases the calibration curve became a saturation curve at higher concentration values), *k*_*n*_ is the coverage factor selected as 2.96 using the standard deviation of 10 consecutive blank signals [22]. The LOD values ranged from 22 pptv (125 pg/l) for 3-octanone to 193 pptv (744 pg/l) for 3-methyl-butanal. The limit of quantification (LOQ) is defined as 3 × LOD. RSDs were calculated on the basis of consecutive analyses of 10 independent standard mixtures exhibiting concentrations close to the means of the observed levels in the actual samples. The RSDs range was 0.86–5.80%.

### Characteristic trends of the detected MVOCs during cultivation

#### Detected alcohols

##### Ethanol

**Fig. 2:**
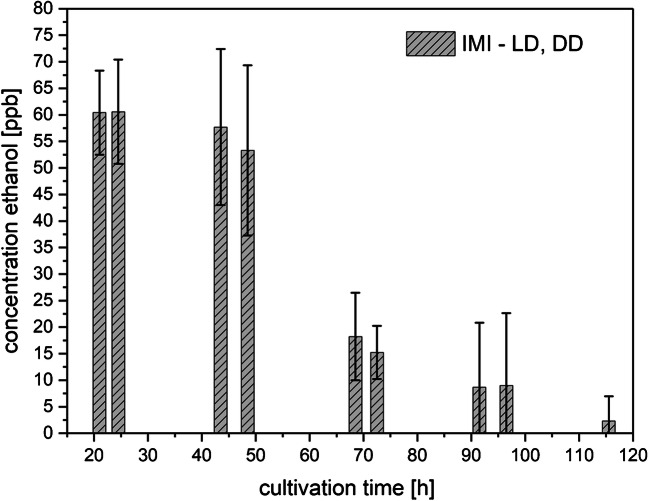
Concentrations of ethanol (means ± SD; *n* = 8) released to the culture headspace as a function of cultivation time

High ethanol release, over 100 ppbv, was observed for axenic cultures of the phytopathogen *F. oxysporum* (Fo) and for co-cultures upon incubation in complete darkness (DD) or in light-dark (LD). Since the reactant ions are consumed over 100 ppbv for ethanol, no quantification was possible above this limit. However, no ethanol was detected above the medium (without inoculation of fungi) under LD and DD conditions. The mycoparasitic fungus *T. atroviride* (IMI) grown in axenic culture under DD or LD conditions emitted ~ 60 ppbv ethanol at the beginning of the cultivation period, which decreased slowly during the course of 5 days of investigation (Fig. 2). Results from cultures grown in DD or LD were averaged since concentrations were generally similar for the eight samples, as indicated by the standard deviations.

##### 1-Propanol

**Fig. 3:**
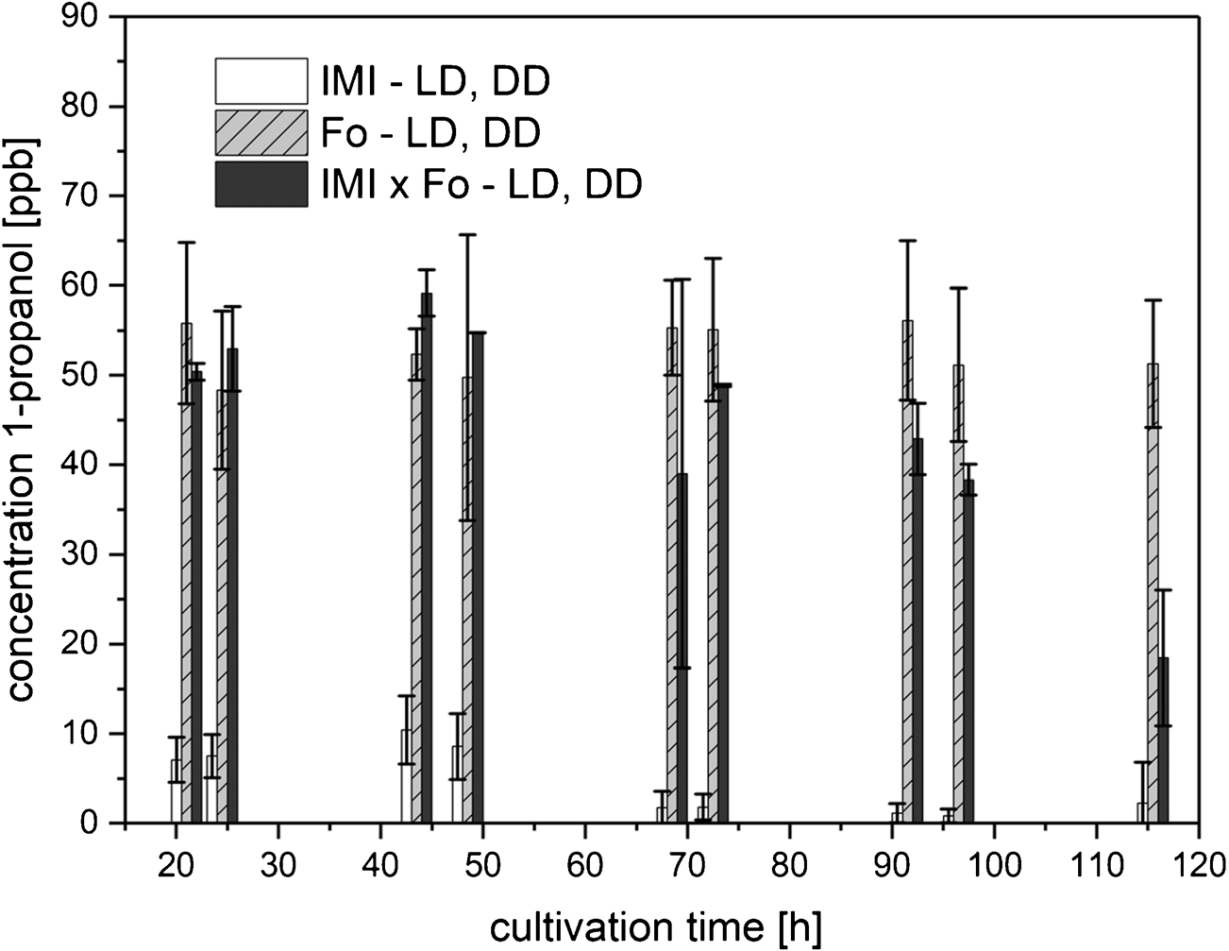
Amounts of 1-propanol (means ± SD; *n* = 8) released to the culture headspace as a function of cultivation time

1-Propanol was produced by both fungi irrespective of the light conditions (Fig. 3), therefore data from LD and DD treatment are averaged. However, the release of 1-propanol into the headspace differed in a strain-specific way. IMI in axenic culture released up to 10 ppbv and axenically grown Fo as well as the co-culture (IMI x Fo) emitted up to 60 ppbv along the cultivation period. The production at the beginning of the cultivation period was higher in co-cultures compared with axenic growth of Fo. However, towards the end of cultivation, when the mycoparasite IMI overgrew Fo, the amount of 1-propanol in the co-cultures dropped to about 30% of the initial value.

##### 2-Methyl-propanol, 2-methyl-butanol, and 3-methyl-butanol

**Fig. 4:**
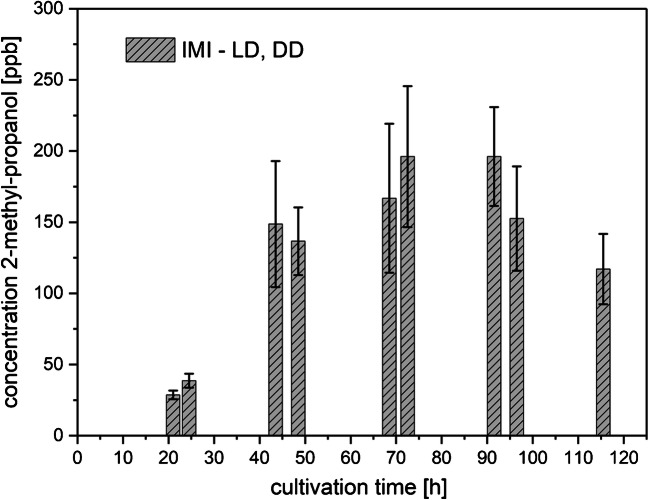
Amounts of 2-methyl-propanol (means ± SD; *n* = 8) released to the culture headspace as a function of cultivation time

2-Methyl-propanol and the two alcohol isomers, 2-methyl-butanol and 3-methyl-butanol, were released by Fo into the headspace in such a high concentration that the reactant ion peak (RIP) was completely depleted (MVOC concentrations higher than 500 ppbv). The production was independent of light exposure and remained unchanged during the whole period of measurements. The same effect could be observed for the co-cultures, while very low levels of 2-methyl-butanol (11.5 ± 4.7 ppbv in LD and 11.3 ± 7.2 ppbv in DD) and of 3-methyl-butanol (9.7 ± 3.0 ppbv both in LD and in DD) were detected in axenic IMI cultures during the whole cultivation time. 2-methyl-propanol release increased in the headspace of IMI axenic cultures until the third day of cultivation reaching around 200 ppbv (Fig. 4) and decreased again towards the end of the cultivation time. Results under LD and DD conditions were averaged since concentrations were similar for the eight samples, as indicated by the standard deviations.

##### 1-Octen-3-ol

**Fig. 5:**
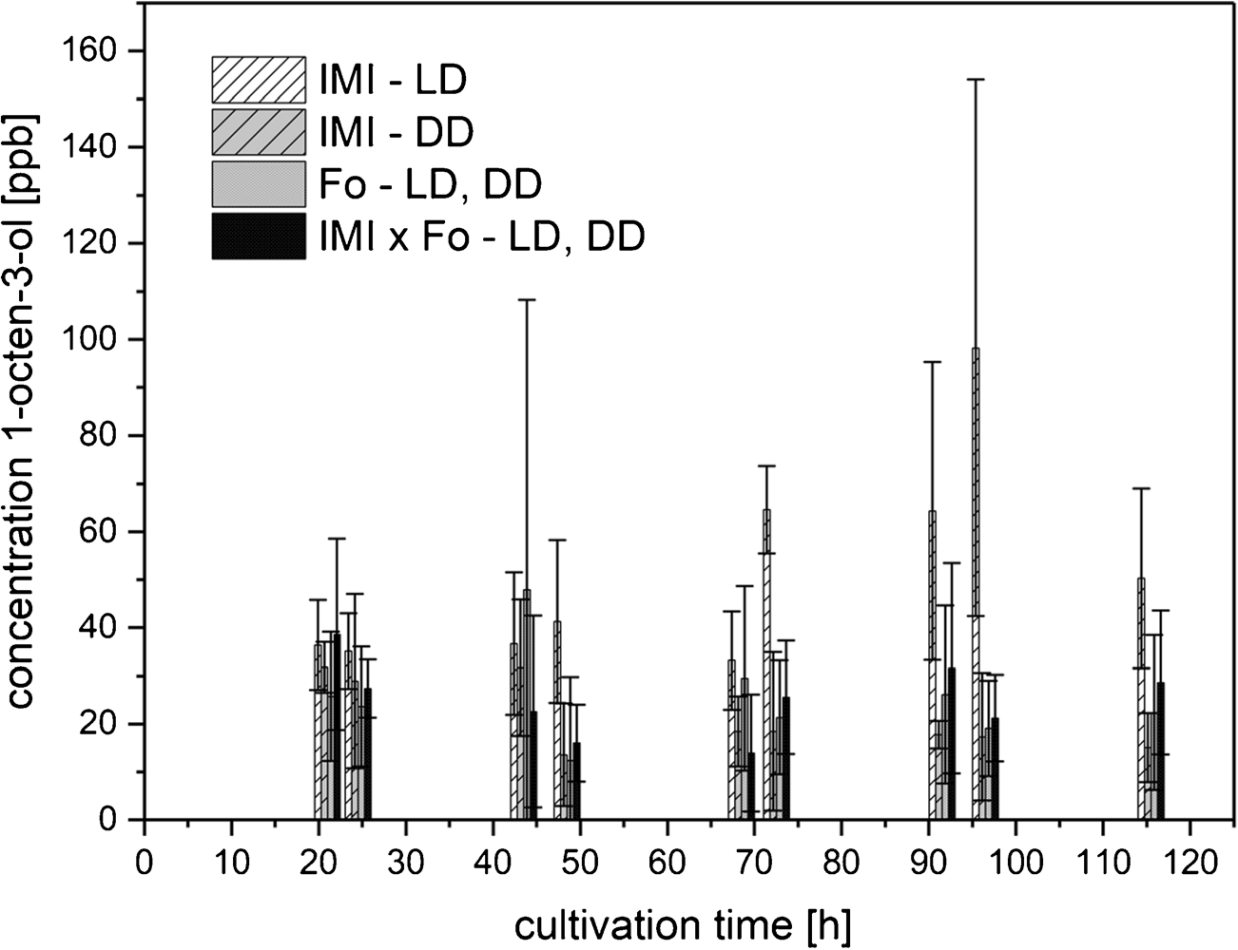
Amounts of 1-octen-3-ol (means ± SD; *n* ≥ 4) released to the culture headspace as a function of cultivation time

An almost constant production of 1-octen-3-ol by both axenic IMI and Fo cultures as well as co-cultures (IMI × Fo) was observed (Fig. 5). The highest 1-octen-3-ol levels of above 100 ppbv were measured in axenic IMI cultures at 96.5 h of cultivation in the presence of light (LD).

#### Detected ketones

##### Acetone

No clear trend was observed for acetone emission during the cultivation period. However, the emitted amounts in average differed between Fo (average concentration 75.8 ± 37.1 ppbv in DD and 180.9 ± 85.4 ppbv in LD) and IMI (36.6 ± 18.6 ppbv in DD and 35.3 ± 17.9 ppbv in LD) as well as co-cultures (80.6 ± 28.7 ppbv in DD and 71.0 ± 51.1 ppbv in LD). Interestingly, light exposure led to a 2-fold increase of acetone emission in axenic cultures of Fo compared with cultivation in DD, while no such effect was observed for IMI (data not shown).

##### 2-Pentanone

**Fig. 6:**
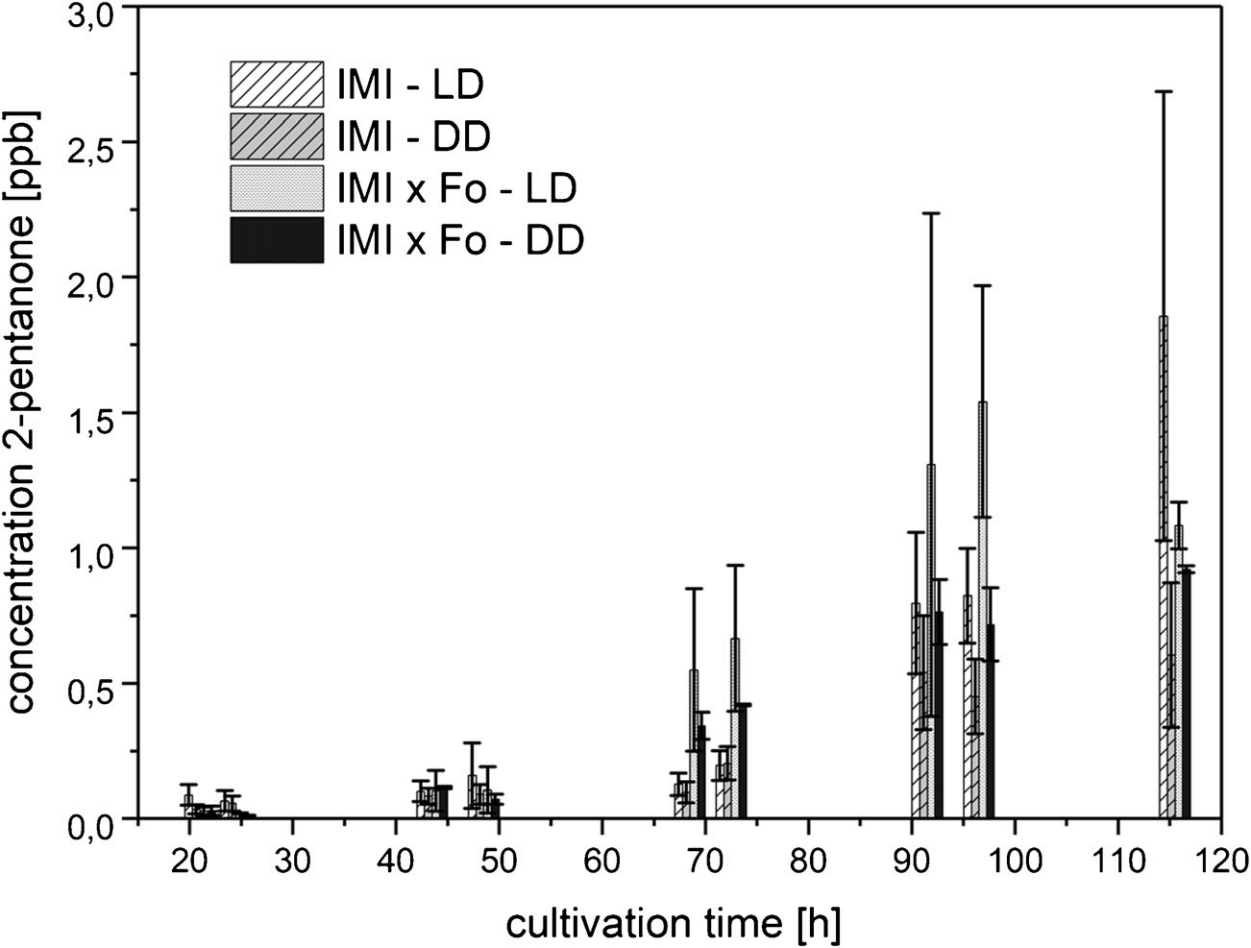
Amounts of 2-pentanone (means ± SD; *n* = 4) released to the culture headspace as a function of cultivation time

**Fig. 7:**
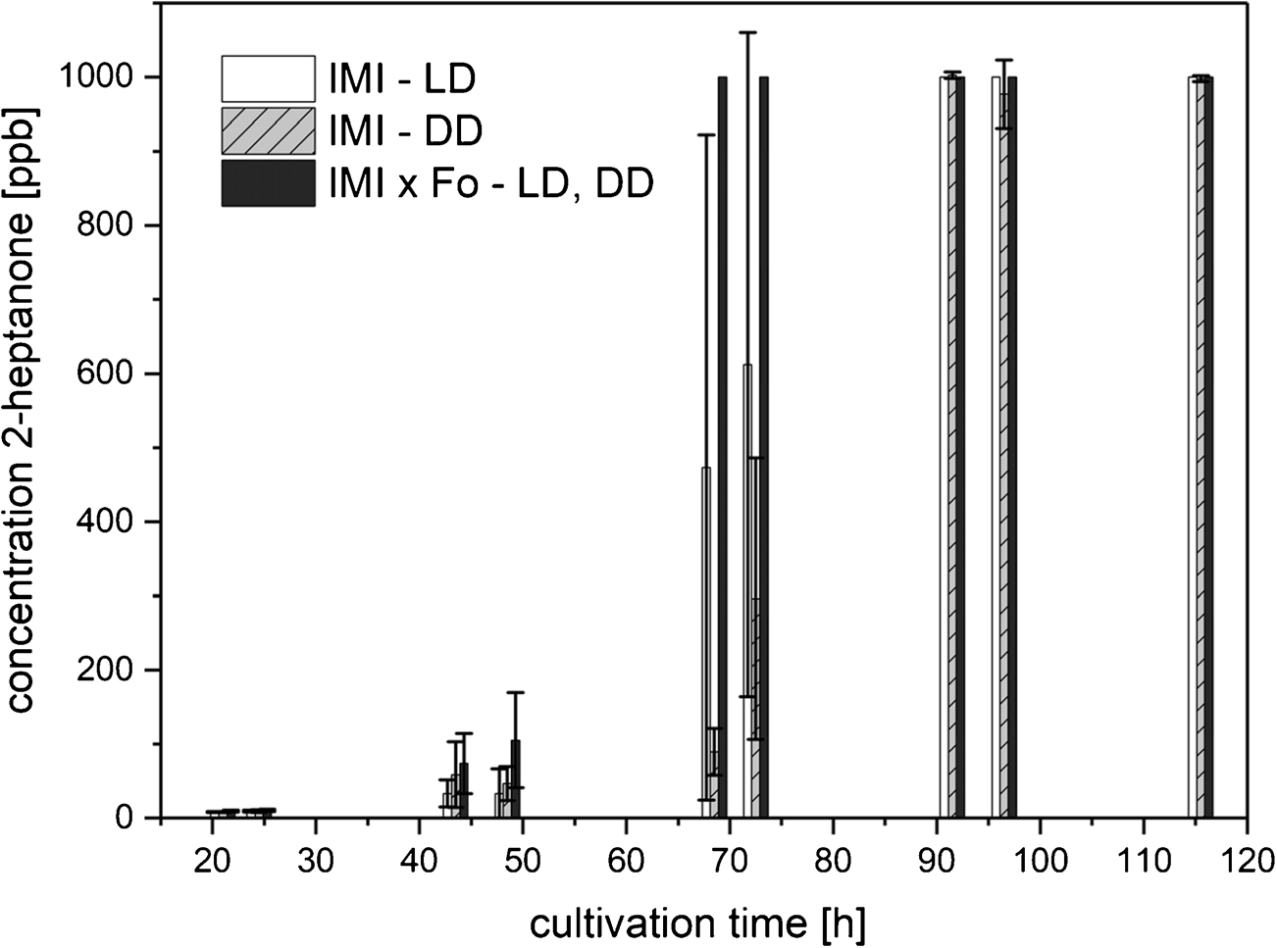
Amounts of 2-heptanone (means ± SD; *n* ≥ 4) released to the culture headspace as a function of cultivation time

Emitted 2-pentanone levels were extremely low, being between 0 and 3 ppbv and hence well within the dynamic range of the GC-IMS device (Fig. 6), thus allowing a more exact quantification compared with 2-heptanone. While Fo released no 2-pentanone to the headspace, IMI and the co-cultures produced this substance with different rates depending on the light regime. Higher 2-pentanone levels were detected in LD-grown cultures compared with cultures grown in DD. Similar to 2-heptanone (Fig. 7), co-cultivation triggered an earlier and higher 2-pentanone biosynthesis than axenic cultivation especially upon LD incubation.

##### 2-Heptanone

Strain IMI emitted over 500 ppbv of 2-heptanone, with a higher increase in 2-heptanone production in the presence of light compared with darkness. The difference in the emitted amounts of 2-heptanone between LD and DD conditions after 70 h of cultivation reached levels of approximately 400 ppbv (with very high standard deviations of the measured values, since at this concentration the reactant ions are almost depleted) (Fig. 7). Towards the end of the cultivation period, no difference between the cultures incubated in LD and DD could be detected. This was because the upper concentration limit of the GC-IMS was reached. In contrast, 2-heptanone could not be detected in the headspace of Fo cultures during the whole cultivation period irrespective of the light conditions. Co-cultivation triggered the production of 2-heptanone already after 40 h of cultivation; after 68.5 h, the concentrations exceeded 400 ppbv in both LD and DD conditions.

##### 3-Octanone

**Fig. 8:**
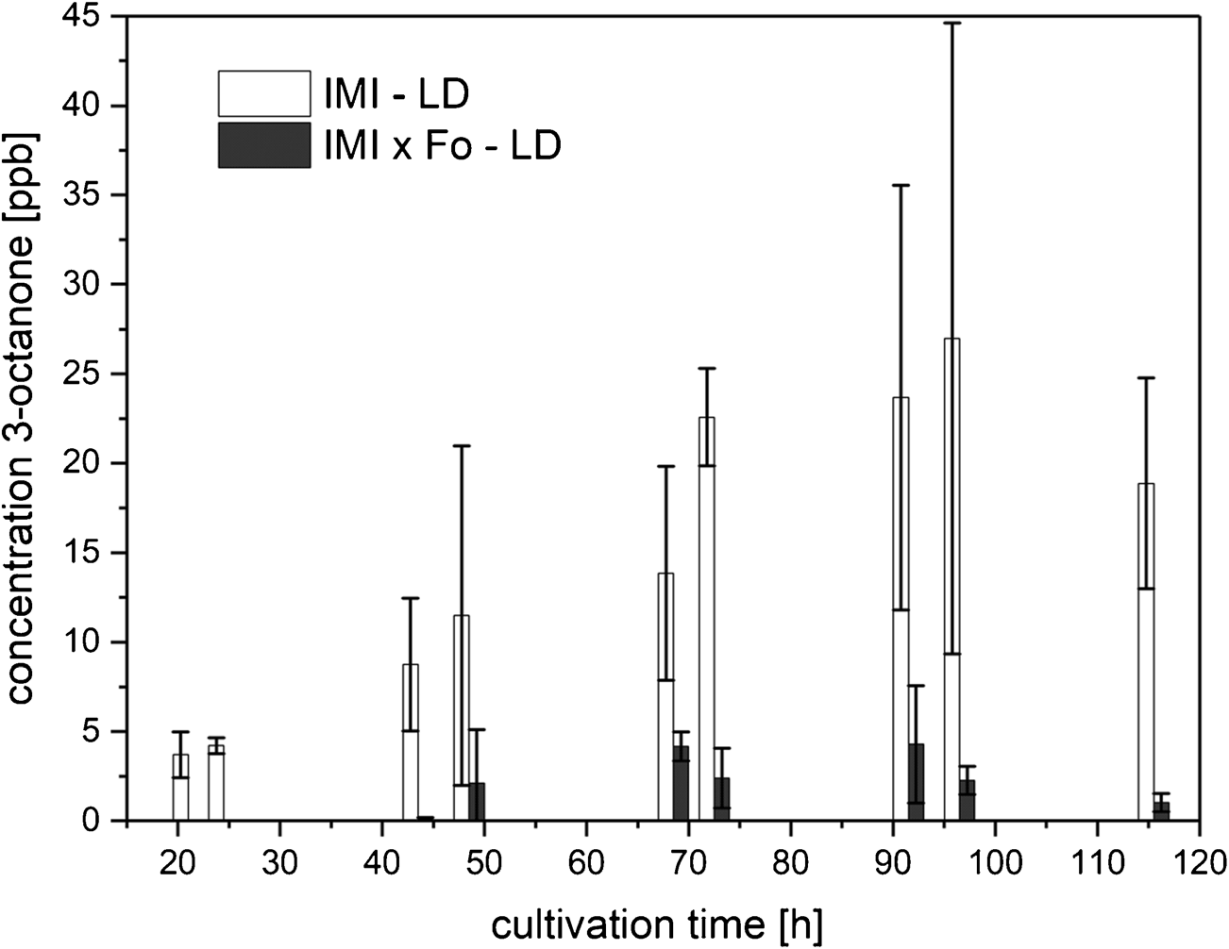
Amounts of 3-octanone (means ± SD; *n* = 4) released to the culture headspace as a function of cultivation time

3-Octanone was detected only in the headspace of light-exposed axenic cultures of IMI as well as during the mycoparasitic interaction (Fig. 8), while no 3-octanone was produced upon cultivation in DD or in axenic culture of Fo. Upon growth in LD conditions, 3-octanone levels emitted by IMI in axenic culture, as well as in co-culture with Fo, increased from ~ 5 ppbv to around 30 ppbv and peaked at 96 h of cultivation. However, upon axenic cultivation, IMI emitted 5–6-fold higher amounts of 3-octanone to the headspace compared with co-cultivation (Fig. 8) suggesting an inhibitory effect of *F. oxysporum* on 3-octanone biosynthesis by IMI.

##### 2-Octanone

**Fig. 9:**
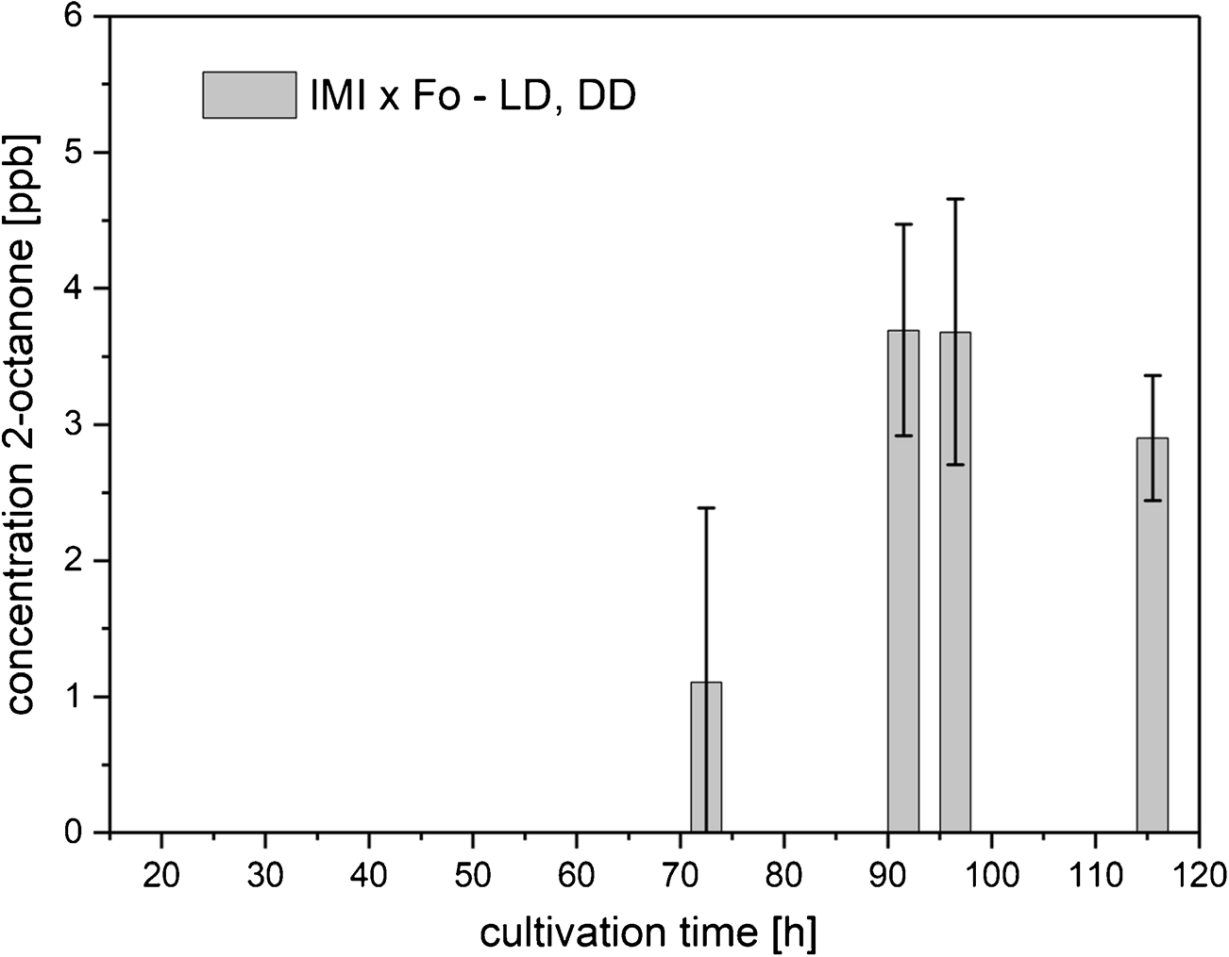
Amounts of 2-octanone (means ± SD; *n* = 8) measured in the headspace of co-cultures of *F. oxysporum* and *T. atroviride* (IMI x Fo) as a function of cultivation time

2-Octanone was exclusively detected during co-cultivation of Fo and IMI from 72.5 h of the cultivation period on, under both LD and DD conditions (Fig. 9), while no 2-octanone could be observed in the axenic cultures of both strains. The emission of 2-octanone solely in co-cultivation after 3 days of growth could indicate that the biosynthesis of this compound is triggered by the direct mycoparasitic interaction between either species or the overgrowth of the host Fo by the mycoparasite IMI.

#### Aldehydes

##### 3-Methyl-butanal

**Fig. 10:**
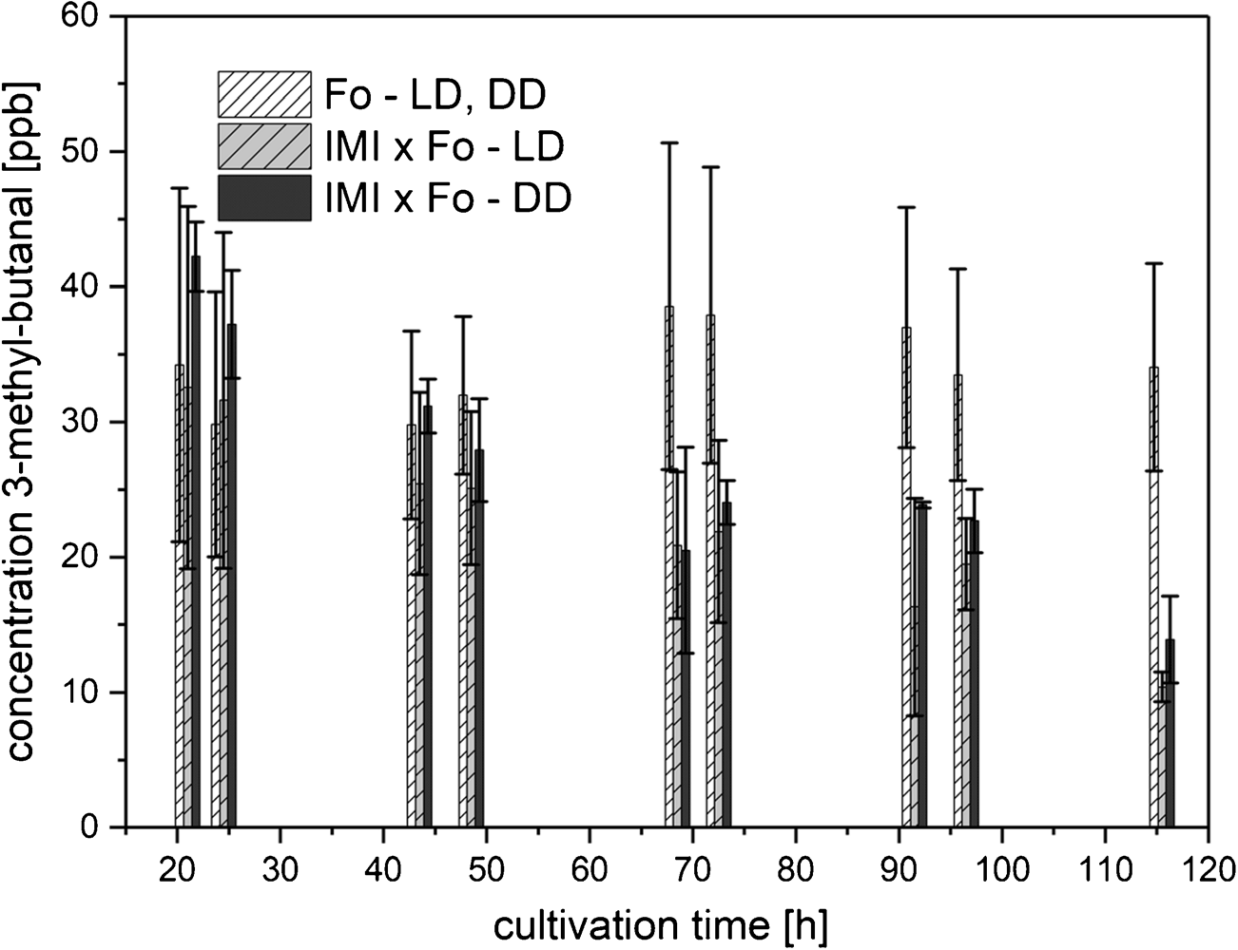
Amounts of 3-methyl-butanal (means ± SD; *n* ≥ 4) released to the culture headspace as a function of cultivation time

A rather constant production of 3-methyl-butanal by Fo with levels around 30 ppbv (Fig. 10) was observed for the whole cultivation period under LD as well as DD conditions. No 3-methyl-butanal was detected in the headspace of axenic grown IMI cultures, irrespective of the applied light regime. Upon co-cultivation, the amount of 3-methyl-butanal slightly decreased from 35 ppbv to about 10 ppbv at the end of cultivation, which could be explained by Fo being overgrown and lysed by the mycoparasitic non-producer IMI.

#### Esters

##### Ethylacetate and 1-butanol-3-methyl-acetate

**Fig. 11:**
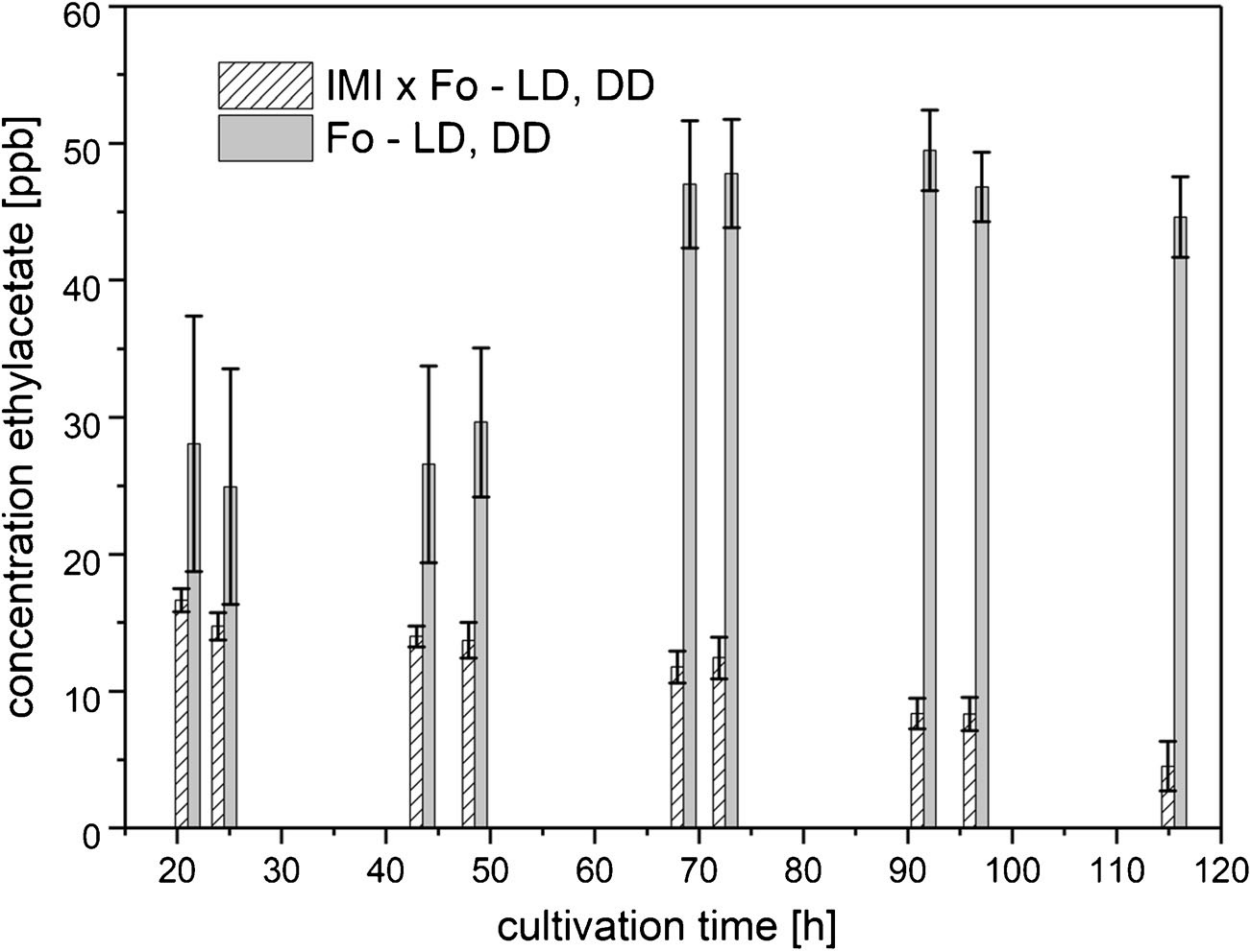
Amounts of ethylacetate (means ± SD; *n* = 8) released to the culture headspace as a function of cultivation time

Ethylacetate was detected only in the headspace of axenic Fo cultures as well as in its direct interaction with IMI (Fig. 11) under both light regimes, while no ethylacetate was released to the headspace by axenic IMI cultures. Starting at around 30 ppbv at the first sampling time point, the values slightly increased in the axenic Fo cultures until the third day of measurement. In the headspace of the co-cultivation of Fo with IMI, the release of ethylacetate already was around 30% lower (around 20 ppbv) from the beginning of the sampling period on and the average concentrations further decreased until the end of cultivation. This decrease could indicate that the biosynthesis of this compound is repressed by IMI-specific air-transmitted signals already at the beginning of the cultivation and further decreased by the overgrowth and lysis of Fo by the mycoparasite IMI.

Isoamylacetate (or 1-butanol-3-methyl-acetate) could be detected only in the headspace of the axenic Fo cultures at very low levels, 1.8 ± 0.4 ppbv in LD and 1.6 ppbv ± 0.4 ppbv in DD during the whole cultivation period. In co-cultures, at the first and second sampling points (after 21 h and 24.5 h), concentrations below 1 ppbv could be measured, but thereafter no characteristic peaks were visible in the GC-IMS spectra anymore.

## Discussion

MVOCs play important roles within the natural habitat. In fungi, they regulate developmental processes like conidiation, exhibit antibacterial and antifungal properties, and contribute to the successful establishment of the fungus in its ecological niche and its interactions with other organisms. The qualitative and quantitative analysis of MVOCs using sophisticated analytical devices with good selectivity to interrogate a culture’s headspace in real or near-to-real time aids in the evaluation of their role and helps to understand their regulatory function at the metabolic level. Until now, GC-MS has been applied as the gold standard for metabolic studies involving molds for the analysis of volatiles. However, this technique requires sample enrichment (mainly solid-phase micro extraction, SPME or adsorbent tubes) [23, 24], which extends the already longer time (in average 40–50 min) for analysis and might lead to loss or changed concentrations of some VOCs depending on the adsorption characteristics on the selected sorbent materials. Although GC-MS analysis is needed, because it is the optimal screening tool for unknown compounds with identification being aided through the use of the integrated NIST database, GC-IMS is ideal for the monitoring of selected VOCs to follow their changes in concentration in near-to-real time through a direct and fast sampling and selective analysis of volatile metabolites. This permits the tracking of metabolic changes that occur on the timescale of minutes, which opens up new areas of study for the biosciences.

GC-IMS detection sensitivity is in the hundreds of pptv, making it ideal for MVOC quantification in the low ppbv range. Since ion mobility spectrometry allows a sensitive analysis of compounds with high gas-phase basicity, this study was focused on the detection of mainly polar compounds such as alcohols, ketones, aldehydes, and esters in the headspace of fungal cultures. A limitation of our GC-IMS is the comparatively small dynamic range due to the consumption of the reactant ions (protonated water clusters), resulting in an upper quantification level of several hundreds of ppbv for the abovementioned compounds.

It is well known that the biosynthesis of VOCs from plants is regulated by various biotic as well as abiotic environmental cues [25, 26]. Contrary to this, knowledge on the triggers influencing and affecting MVOC biosynthesis in fungi is more limited [27]. In this study, we could prove that after establishing standardized cultivation, sampling, and analysis conditions and a respective component library, two fungal species and their co-cultures emitted characteristically differing volatile profiles. Furthermore, light- and cultivation time-dependent effects in MVOC release have been detected. The influence of light exposure was especially clear for the ketones 2-pentanone, 2-heptanone, and 3-octanone, and for the alcohol 1-octen-3-ol. We detected a strongly light-dependent emission of 3-octanone by *T. atroviride* IMI206040, a volatile that was not be detected in dark-grown cultures, and most probably caused by the lack of auto-induction of conidiogenesis in this strain upon completely dark growth conditions [20].

MVOCs have a role in diverse regulation processes independent of their chemical class. Concerning the compounds detected in our study, the alcohol 1-octen-3-ol has been reported to act as an insect attractant, exhibit antimicrobial properties, inhibit the germination and growth of plant seedlings, and induce systemic resistance against pathogens in plants [28–30]. Both, 1-octen-3-ol and the ketone 3-octanone, are involved in the concentration-dependent regulation of conidiogenesis in fungi [31–33]. The ketone 2-heptanone has been reported to act as allomone, thereby attracting insects and nematodes towards the prey [34, 35] and the alcohol 2-methyl-propanol and the ester ethylacetate are known to lure fungivorous nematodes and collembolans to the hyphal network and dispersal units [36, 37].

Interestingly, the alcohols 2-methyl-butanol, 3-methyl-butanol, and 2-methyl-propanol were produced in significantly higher amounts by *F. oxysporum* than by *T. atroviride* upon axenic cultivation. These fusel alcohols are known to contribute to the characteristic flavors in various fermented food products [38] and in edible mushrooms, such as truffles [39]. Furthermore, they have been described to be involved in quorum-sensing mechanisms and in morphogenesis in yeast [40]. Fusel alcohols derive from the catabolism and biosynthesis of branched-chain amino acids via the Ehrlich pathway [41] whose regulation is to date unclarified—a connection to nitrogen limitation most probably plays a role [40]. In the interaction of pathogenic fungi, these alcohols as well as mixtures of *Fusarium* MVOCs have been observed to inhibit plant seed germination and affect plant growth [39, 42] as well as to act as antifungal [43] and nematicidal [44] agents. The esters ethylacetate and 1-butanol-3-methylacetate, which are generated by the conversion of alcohols and acetyl CoA via versatile, partially unclarified biosynthetic pathways [45] in microbes, were released exclusively by *F. oxysporum*. Interestingly, the mycoparasitic interaction of *T. atroviride* with *F. oxysporum* was characterized by the biosynthesis of 2-octanone, while this metabolite was absent in the axenic cultures of both fungi. A recent study reported that deletion of the Hda2 histone deacetylase-encoding gene in *T. atroviride* gave rise to the production of this MVOC [46]. This indicates that *F. oxysporum* triggers the alleviation of repressing chromatin structures in the mycoparasite and that 2-octanone may contribute to the successful antagonism of this host fungus. Co-cultivation, especially under light exposure, led to enhanced levels of the ketones 2-pentanone and 2-heptanone compared with axenic cultivation. Fungal ketone biosynthesis is to date not well characterized. A de novo biosynthesis via β-oxidation of fatty acids was suggested for the methyl ketone 2-pentanone in *Penicillium roqueforti* [47] and in *Saccharomyces cerevisiae* [48]. In our study, three ketones, 2-heptanone, 2-pentanone, and 3-octanone, which might derive from β-oxidative conversion of specific fatty acids [49], were solely emitted by the mycoparasite *T. atroviride* and in co-cultivation. The enriched release of 2-heptanone and 2-pentanone during the later phases of the co-cultivation compared with axenic cultivation of *T. atroviride* could indicate an important role of these metabolites in mycoparasitism. In contrast, co-cultivation led to reduced levels of ethylacetate, 1-butanol-3-methyl-acetate, and 3-octanone compared with the axenic cultures.

In conclusion, our results highlight that the GC-IMS approach is a valuable analytical tool for monitoring identified metabolite patterns for chemotaxonomic applications, and that it is highly applicable to monitor and characterize the metabolic state of different cultivation conditions in solid fungal cultures. Hence, we propose that the application of GC-IMS has considerable potential for on-line monitoring of liquid fermentations in bioreactors.
